# Quantification of the Contact Area at the Head-Stem Taper Interface of Modular Hip Prostheses

**DOI:** 10.1371/journal.pone.0135517

**Published:** 2015-08-17

**Authors:** Florian Witt, Julian Gührs, Michael M. Morlock, Nicholas E. Bishop

**Affiliations:** 1 Institute of Biomechanics, TUHH Hamburg University of Technology, 21073 Hamburg, Germany; 2 Faculty of Life Sciences, HAW Hamburg University of Applied Sciences, Ulmenliet 20, 21033 Hamburg, Germany; North Carolina A&T State University, UNITED STATES

## Abstract

Corrosion of modular taper junctions of hip implants may be associated with clinical failure. Taper design parameters, as well as the intraoperatively applied assembly forces, have been proposed to affect corrosion. Fretting corrosion is related to relative interface shear motion and fluid ingress, which may vary with contact force and area. It was hypothesised in this study that assembly forces modify the extent and distribution of the surface contact area at the taper interface between a cobalt chrome head and titanium stem taper with a standard threaded surface profile. Local abrasion of a thin gold coating applied to the stem taper prior to assembly was used to determine the contact area after disassembly. Profilometry was then used to assess permanent deformation of the stem taper surface profile. With increasing assembly force (500 N, 2000 N, 4000 N and 8000 N) the number of stem taper surface profile ridges in contact with the head taper was found to increase (9.2±9.3%, 65.4±10.8%, 92.8±6.0% and 100%) and the overall taper area in contact was also found to increase (0.6±0.7%, 5.5±1.0%, 9.9±1.1% and 16.1±0.9%). Contact was inconsistently distributed over the length of the taper. An increase in plastic radial deformation of the surface ridges (-0.05±0.14 μm, 0.1±0.14 μm, 0.21±0.22 μm and 0.96±0.25 μm) was also observed with increasing assembly force. The limited contact of the taper surface ridges at lower assembly forces may influence corrosion rates, suggesting that the magnitude of the assembly force may affect clinical outcome. The method presented provides a simple and practical assessment of the contact area at the taper interface.

## Introduction

High failure rates of metal-on-metal hip joint replacements have been related to wear of the bearing surface, due in particular to edge loading [1]. Clinical failure may also been related to corrosion of the modular taper junction between head and stem, but the mechanisms remain unclear [2–7]. High metal ion concentrations and metal debris have been related to severe local and systemic biological reactions [8–13]. A set of causal factors related to the failure mechanism is still lacking [14,15].

In vivo hip force measurements in patients after total hip arthroplasty have revealed friction moments acting in well-functioning bearings during gait [16]. Joint friction moments have been shown experimentally to increase in conditions of poor lubrication [17]. Joint friction moments under such conditions have been suggested to induce relative motion at the interface between modular components, facilitating fretting corrosion [18]. This mechanism could explain the increasing revision rates of modular metal-on-metal bearings with increasing bearing diameter [19], since the joint friction moment increases with bearing diameter [17]. The hypothesis that failure of the taper junction can be related to compromised joint lubrication has been confirmed for retrieved components, by comparing the material loss from the bearing surface with material loss from the taper junction [18]. However, this association could not be demonstrated exclusively for each retrieval, and neither were there any clear influences of other measurable parameters on taper geometry. This suggests that other factors are involved, that may be related to the surgical procedure, implant variation or patient factors.

Laboratory experiments have demonstrated that the rate of corrosion of a cobalt chrome head on a titanium stem decreases with increasing applied assembly force [20]. It has been shown that such metal corrosion is initiated by the mechanical removal of the protective oxide layer occurring on the reactive metal surface, allowing dissolution of the metal into surrounding fluids [21]. Increased assembly forces could generate sufficient friction resistance at the interface to prevent local fretting, and may also improve sealing of the interface and limit fluid ingress or exchange. Design factors that may modulate this interaction are material and geometry of the taper surfaces and their surface finish. The design of many current stem tapers is based on their use with ceramic heads. Due to their brittle nature, internal ceramic head tapers are designed to seat with the proximal end of the stem in contact with the deepest part of the head taper, to minimise stresses in the ceramic and prevent burst fracture [22]. This is achieved by designing the head taper angle to be greater than that of the stem taper. Furthermore, the surface of the stem taper is designed with a thread-like topography, with the aim of softening the interface and permitting local adaptive deformations, to reduce local stress peaks due to surface irregularities [23].

Although these taper design features were introduced to allow the use of ceramic heads they can now be found on nearly every stem taper, and they are also mated with metal heads. Metal head taper angles have become more similar to those of the stem taper, since metal heads are much tougher than ceramic components. However, it has been shown that the threaded profile of a titanium stem taper can lead to material loss within the cobalt chrome head, with a pattern mirroring the threaded stem taper profile [24]. Another study has related taper corrosion to the actual interface contact area for metal-metal combinations, thereby demonstrating a dependency of corrosion on geometry and surface finish [25]. In the present study it was hypothesized that assembly forces modify the extent and distribution of the surface contact area at the taper interface between a cobalt chrome head and titanium taper with a threaded surface profile. Sputtering of a very thin layer of gold onto the surface using standard equipment was considered as a practical new method to demonstrate interface contact.

## Materials and Methods

### Components

A commercially available and clinically implanted hip stem and metal head design combination were investigated (n = 3). The cobalt chrome head had a diameter of 36 mm, with a 12/14 taper and +1.5 mm offset (M-SPEC, DePuy International Ltd, Leeds, England). The titanium alloy stem had a 12/14 ATM taper (11 mm long) with a threaded surface profile (Corail DePuy International Ltd, Leeds, England). The entire surface of the stem taper was seated within the assembled head.

Taper angles of head and stem were determined using a coordinate measurement machine (Mitutoyo BHN-305, Mitutoyo Deutschland GmbH, Neuss, Germany) and custom surface-fitting software [24]. The measurement probe was equipped with a relatively large 3 mm diameter ruby tip to minimize noise due to the influence of the microscopic surface profile.

### Surface preparation

**Fig 1:**
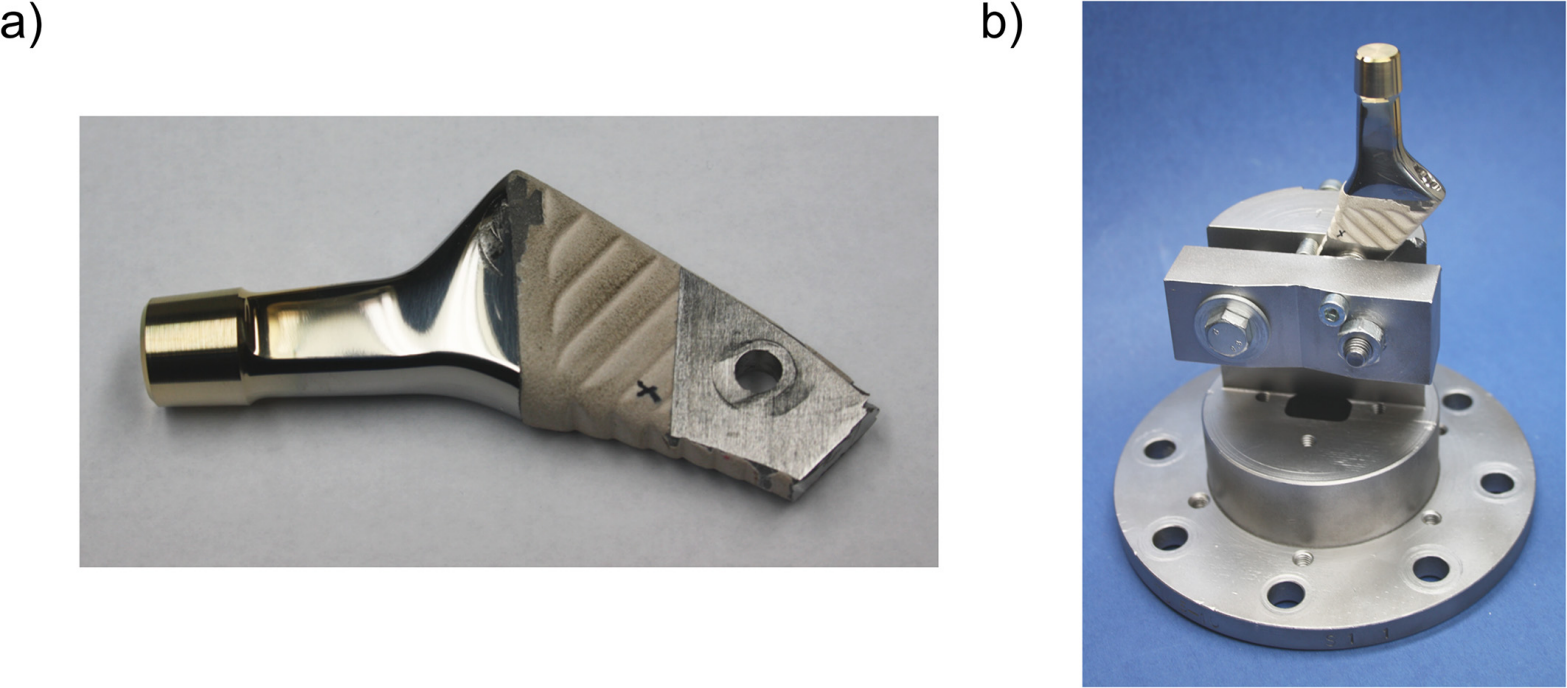
Stem design modifications. (a): Geometrical limitations of the sputtering machine necessitated modification of the stem: It was shortened, parallel planes were milled and a hole was drilled for anchorage. (b): These modifications allowed fixation in a clamping block.

The hip stems were shortened and bolted to a mount (Fig 1), providing reproducible positioning of the taper for surface coating, head assembly and surface measurements. The taper surface was sputtered on the lateral aspect with a 25.3 nm gold layer (SCD 050 sputter coater, BAL-TEC, Scotia, NY, USA; device settings: argon gas, 0.05 mbar working pressure, 40 mA current, 50 mm working distance, sputter time ≈ 150 s). Layer thickness was controlled with a quartz crystal film thickness monitor (EM QSG100, Leica, Wetzlar Germany). The taper contact area was determined after disassembly of the head, by measuring the area of the gold layer removed due to abrasion.

### Assembly and Disassembly

After coating the stem taper surface, each of the three head-stem taper pairs was assembled by quasistatic application of an axial force (0.04 mm/s, according to ISO 7206–10) of consecutive 500, 2000, 4000 and 8000 N to the head (Zwick/Roell Z010, Zwick GmbH & Co. KG, Ulm, Germany). After assembly the head was quasistatically pulled off axially (0.008 mm/s according to ISO 7206–10) using a custom grip with two hooks acting on the flat face of the head (Zwick/Roell Z010, Zwick GmbH & Co. KG, Ulm, Germany). The stem taper surface was analysed before and after each assembly step.

### Surface Analysis

A 700 μm wide strip of the lateral surface of the stem taper was analysed by topographical microscopy (Alicona infinite focus microscope, Alicona Imaging GmbH, Austria). A 20x magnification lens (vertical resolution 250 nm and lateral resolution 1750 nm) was used to map the stem profile from distal to proximal (a sequence of 23 images, which were stitched together). Portable network graphics (“png”) images were exported and transformed into the CMYK colour space (Adobe Photoshop CS5 Extended, Adobe). Using only the Yellow channel, differentiation between regions of gold and no-gold was achieved. Thresholds were initially determined visually and then used throughout in an automated custom-written program (Matlab, R2011a, The MathWorks Inc., Massachusetts, USA). Regions of the surface in which the gold coating was removed were defined as regions of contact.

Regions of permanent deformation of the stem surface profile were determined by superimposing the profile of the stem taper interface after disassembly on the initial stem taper profile and calculating the height reduction of the ridges. Deformation profiles were calculated as the mean of 100 μm wide bands, running from the distal to proximal ends of the taper. Imaging artefacts can be caused by the gold layer due to light reflection. Each profile was therefore filtered for outliers using a threshold of 1 μm. Superimposition of the profiles before and after assembly was achieved by least-squares best-fit of the troughs of the surface profiles (values less than the mean profile height), where the profiles were assumed to remain undeformed. Measurement accuracy was evaluated by repeated positioning and scanning of the same sputtered stem taper (n = 3). The highest deviation along the whole taper length was 0.6 μm.

Linear and exponential regressions were used to relate contact parameters to assembly force. On the basis of Shapiro-Wilk tests for normality, a one-way analysis of variance with a Bonferroni post-hoc test was performed to test the effect of assembly force on contact area and plastic deformation (α = 0.05, PASW Statistics 18, Chicago, IL, 114 USA).

## Results

### Taper angles and surface profile

Stem and head tapers had consistently similar taper angles, resulting in differences of ≤0.01° (Table 1).

**Table 1:** Taper angles. All three component pairs had similar taper angles.

Component	Angle [°]	Component	Angle [°]
Stem 1	5.67	Head 1	5.66
Stem 2	5.67	Head 2	5.67
Stem 3	5.67	Head 3	5.67

**Fig 2:**
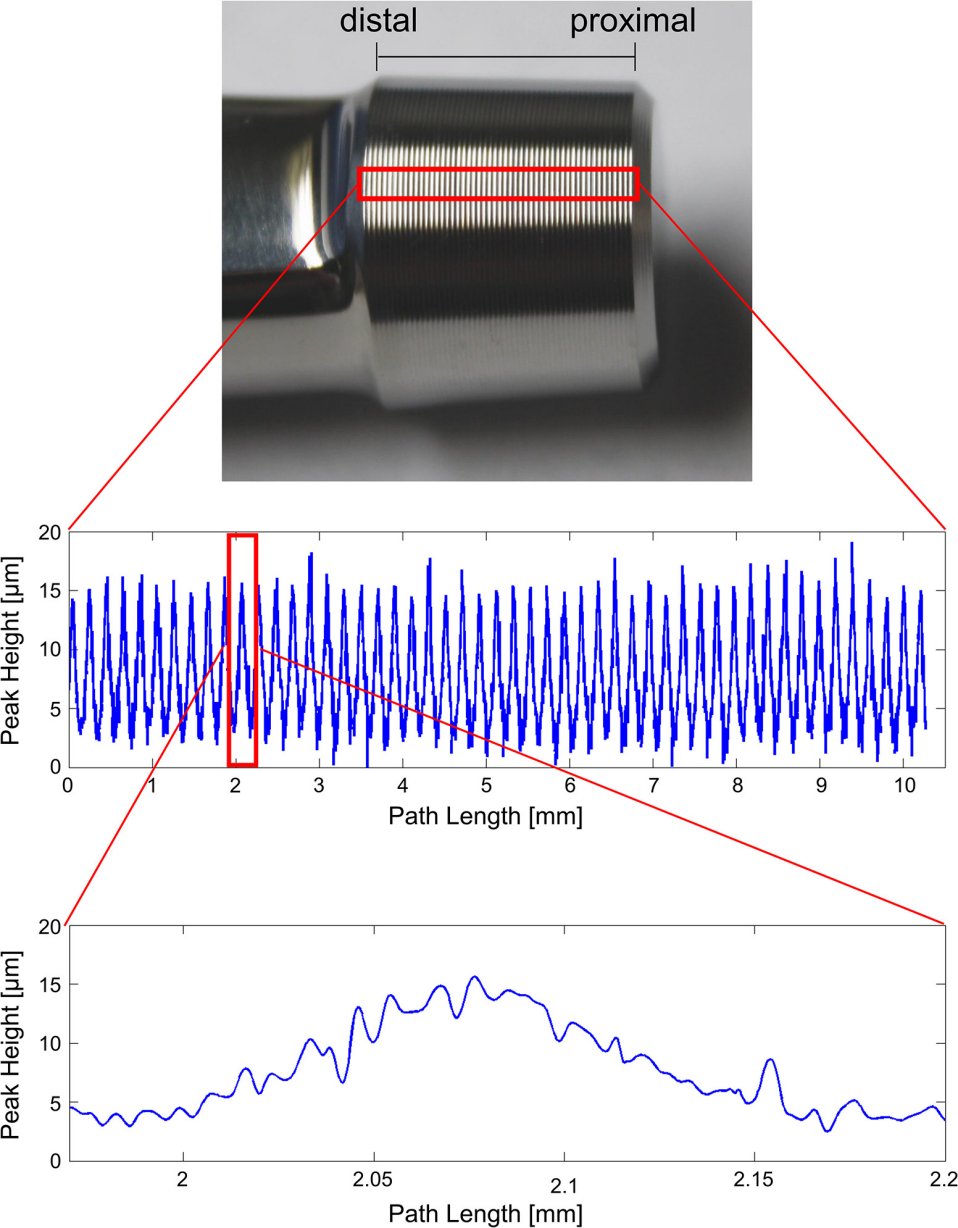
Stem Taper profile. Multiple measurements of the same stem region (red rectangle, top) were performed. A 100 μm wide path from distal to proximal was chosen to obtain a mean profile (middle). It is noted that each profile ridge demonstrates a surface roughness of about an order of magnitude smaller amplitude and period (bottom).

A regular wave profile was observed on the surface of the stem taper, with a wavelength of ~200 μm and an amplitude of ~10 μm (Fig 2). This profile is a right-handed thread spiral, with 51 ridges along the length of the taper. This manufactured surface profile was superimposed by a surface wave of shorter wavelength and lower amplitude (by roughly an order of magnitude).

### Contact regions

**Fig 3:**
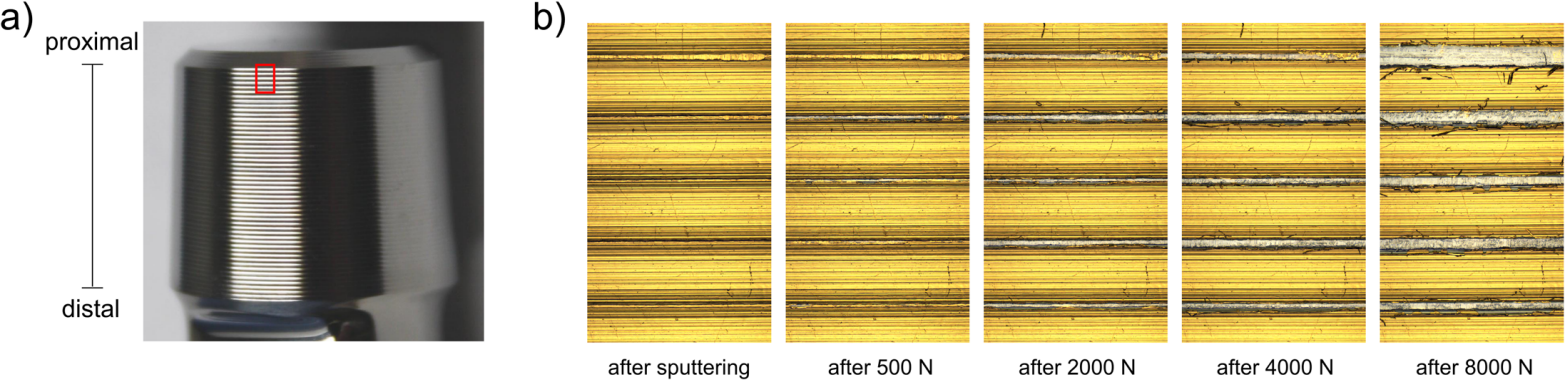
Surface contact. a) The red rectangle on this non-sputtered taper represents the magnified regions in b). b) The first five proximal ridges of stem taper profile #3 are shown. The gold coating is removed from the ridges by shear abrasion during assembly (uncoated silver areas), but remains in the valleys. With increasing assembly force a greater area of the gold coating is removed from the ridges of the threaded surface profile due to increased contact between head and stem.

**Fig 4:**
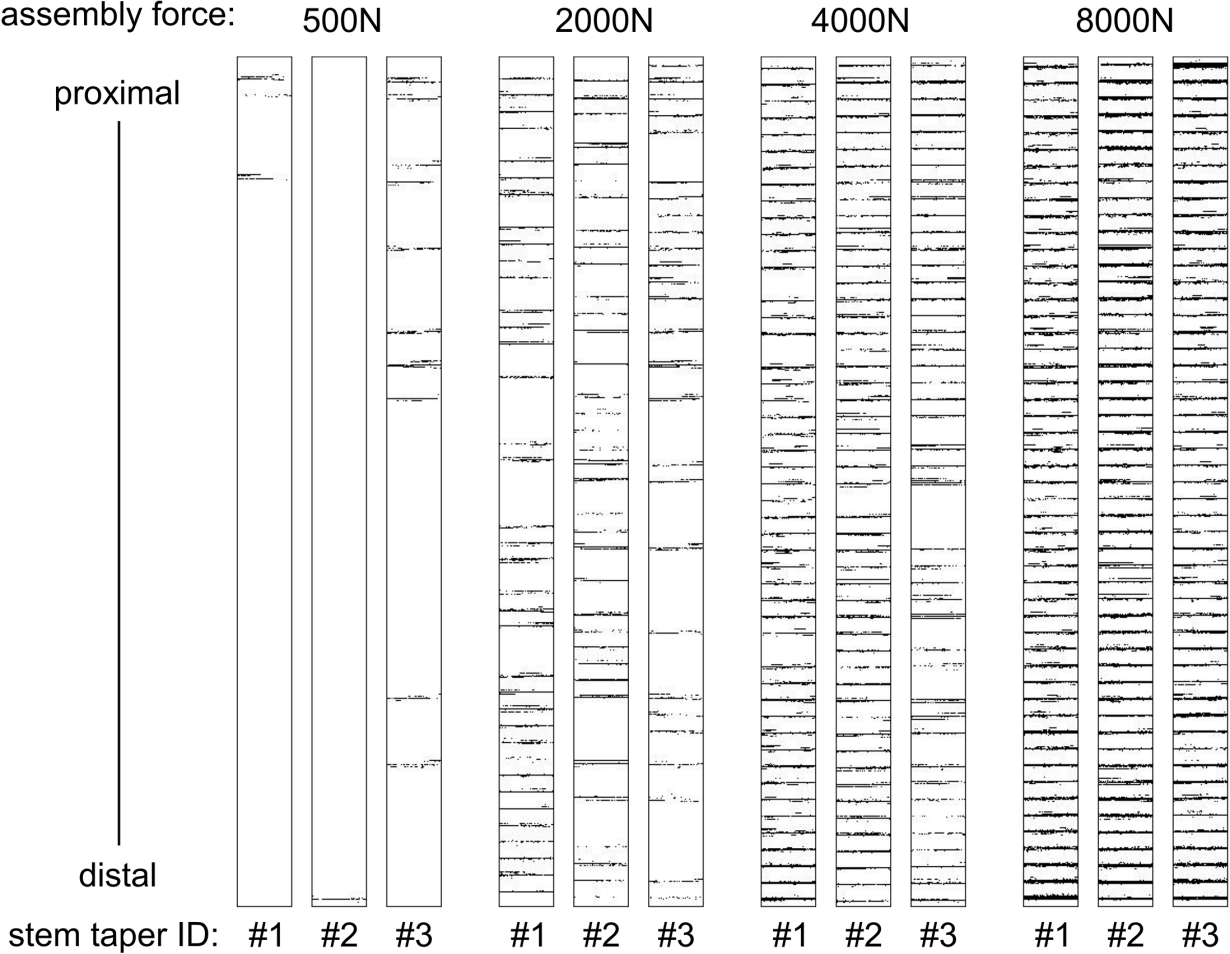
Quantification of surface contact. Black pixels within boxes represent contact areas and contact locations between stem and head component pairs. Profiles are presented for the three tapers under increasing assembly forces.

The extent of surface contact was revealed by abrasion of the gold coating from the stem taper surface (Fig 3). The contact area increased with increasing assembly force, both in terms of the number of ridges demonstrating contact as well as the total area of contact (Fig 4).

**Fig 5:**
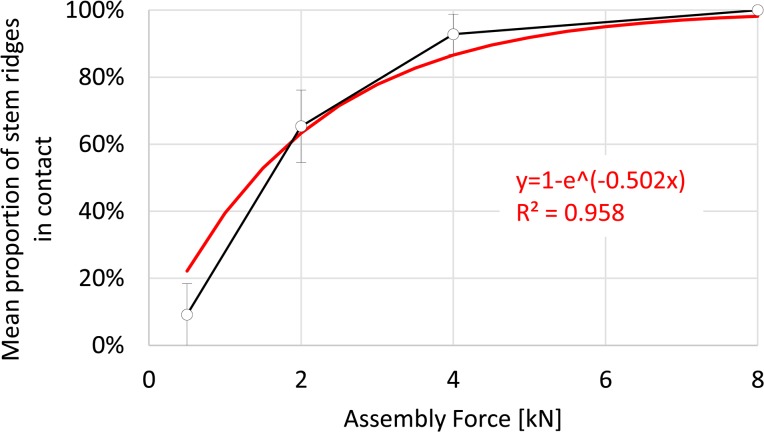
Stem taper ridges in contact. With increasing assembly force an increasing number of stem taper surface ridges came into contact with the head taper surface. For an assembly force of 8000 N all stem taper surface ridges were in contact.

**Fig 6:**
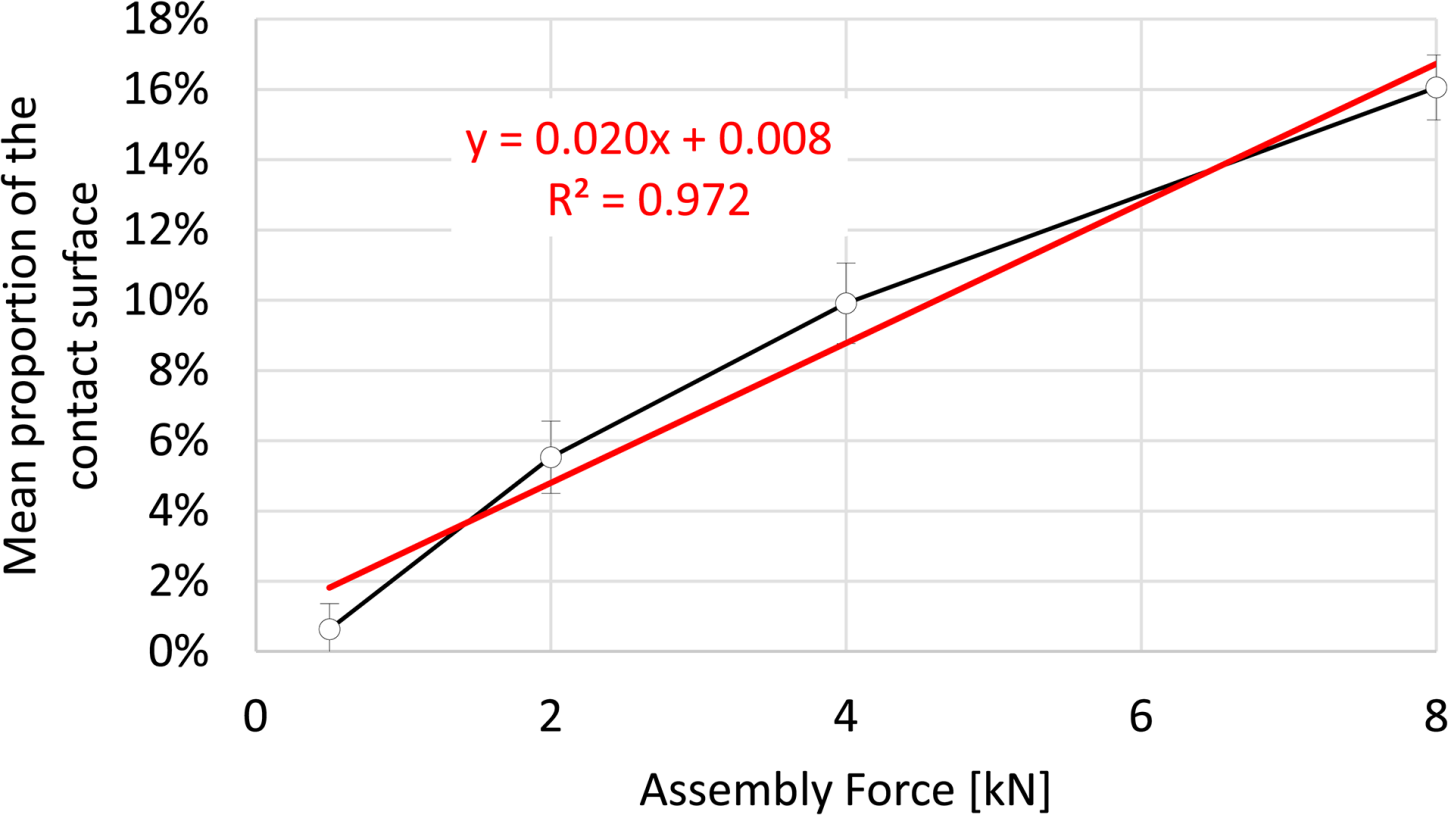
Stem contact area. With increasing assembly force a linear increase of the proportion of the surface in contact was observed.

At the lowest assembly force (500 N) as few as one single ridge was in contact for pair #2, at the distal end of the stem taper (Fig 4). In contrast, only proximal ridges were in contact for pair #1. Contact was distributed over the length of the taper for pair #3. A total of 9.2±9.3% (mean and standard deviation, Fig 5) of stem taper ridges were in contact and resulted in 0.6±0.7% (mean and standard deviation, Fig 6) surface contact. Under an assembly force of 2000 N (this is similar to lower joint force magnitudes measured during walking [26]) 65.4±10.8% of ridges were in contact and resulted in an increase of surface contact to 5.5±1.0%. The distribution of ridges in contact along the length of the taper followed no clear pattern (Fig 4). For 4000 N assembly force both the number of contact ridges and the proportion of surface contact increased to 92.8±6.0% and to 9.9±1.1%, respectively. For 8000 N assembly force, all of the ridges were in contact for all of the samples with 16.1±0.9% of the surface in contact. The increase of the mean proportion of stem taper ridges in contact, with increasing assembly force, followed an exponential function (R^2^ = 0.978, Fig 5) and the mean proportion of the surface in contact demonstrated a linear dependency on assembly force (R^2^ = 0.972, Fig 6). Significant differences in the contact area were observed between each assembly force (p≤0.003).

### Permanent Deformation

**Fig 7:**
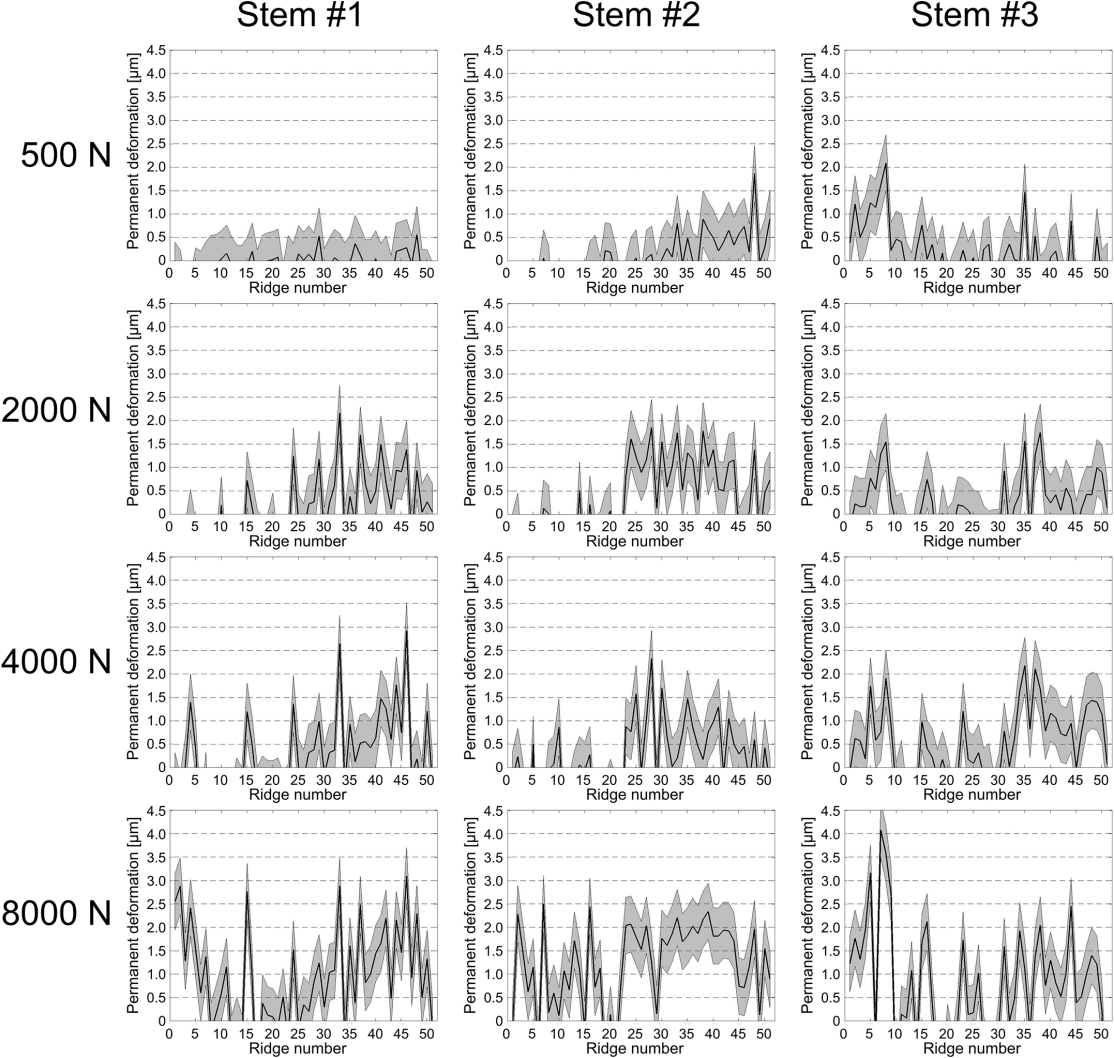
Permanent radial deformation of the stem taper surface profile. The height difference of the stem taper profile between assembled and original condition for each ridge is plotted as a black line, with the measurement accuracy (0.6 μm) in grey.

**Fig 8:**
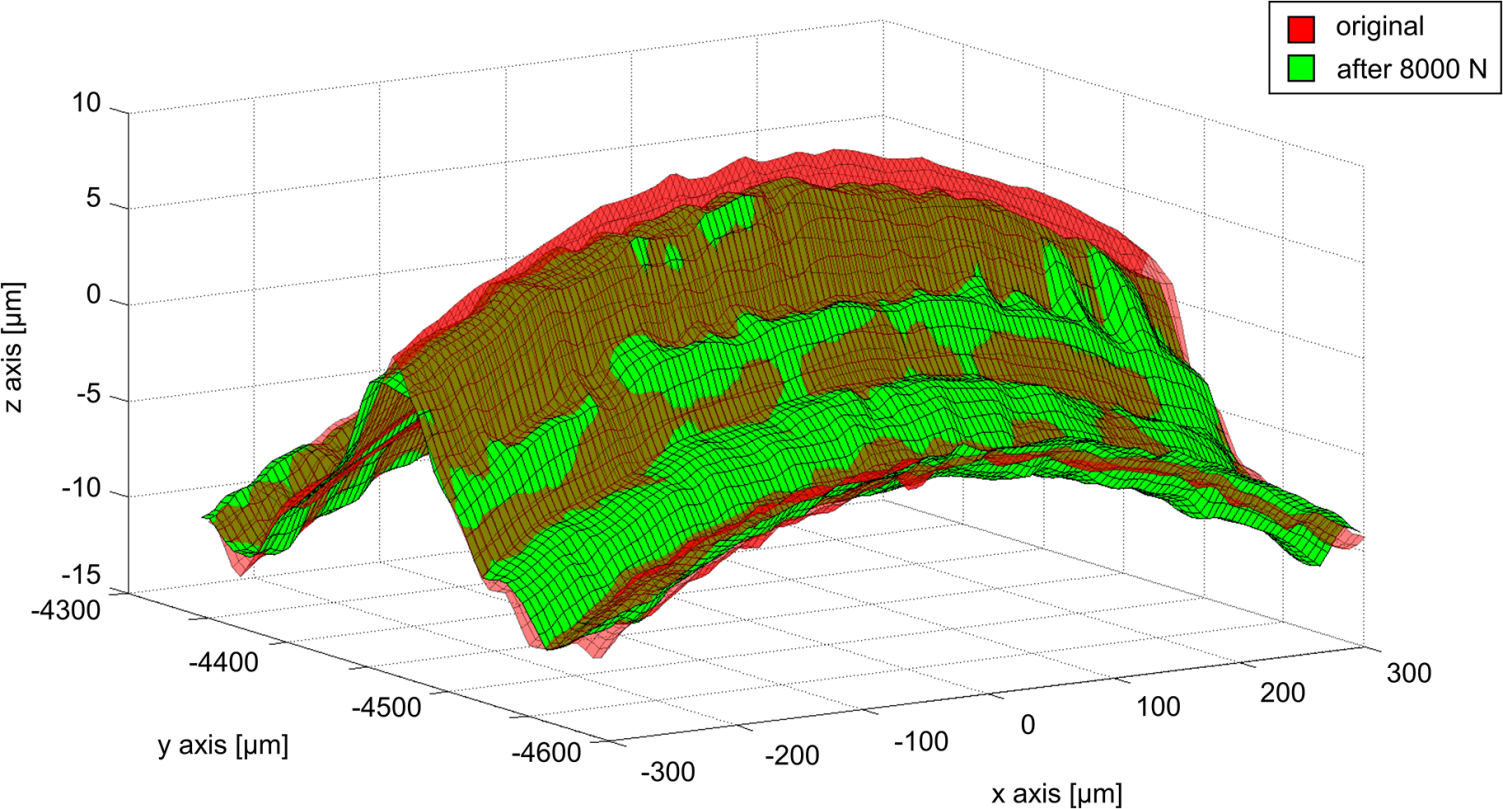
Permanent deformation of a single surface ridge. Regions of permanent deformation of the stem surface profile were identified by superimposing the profile of the stem taper interface after disassembly on the initial stem taper profile. The original shape (red transparent surface) was deformed after assembly with 8000 N (green surface). The ridge height was reduced. Note that the axes are not equally scaled, so that the height and curvature of the wave are exaggerated.

**Fig 9:**
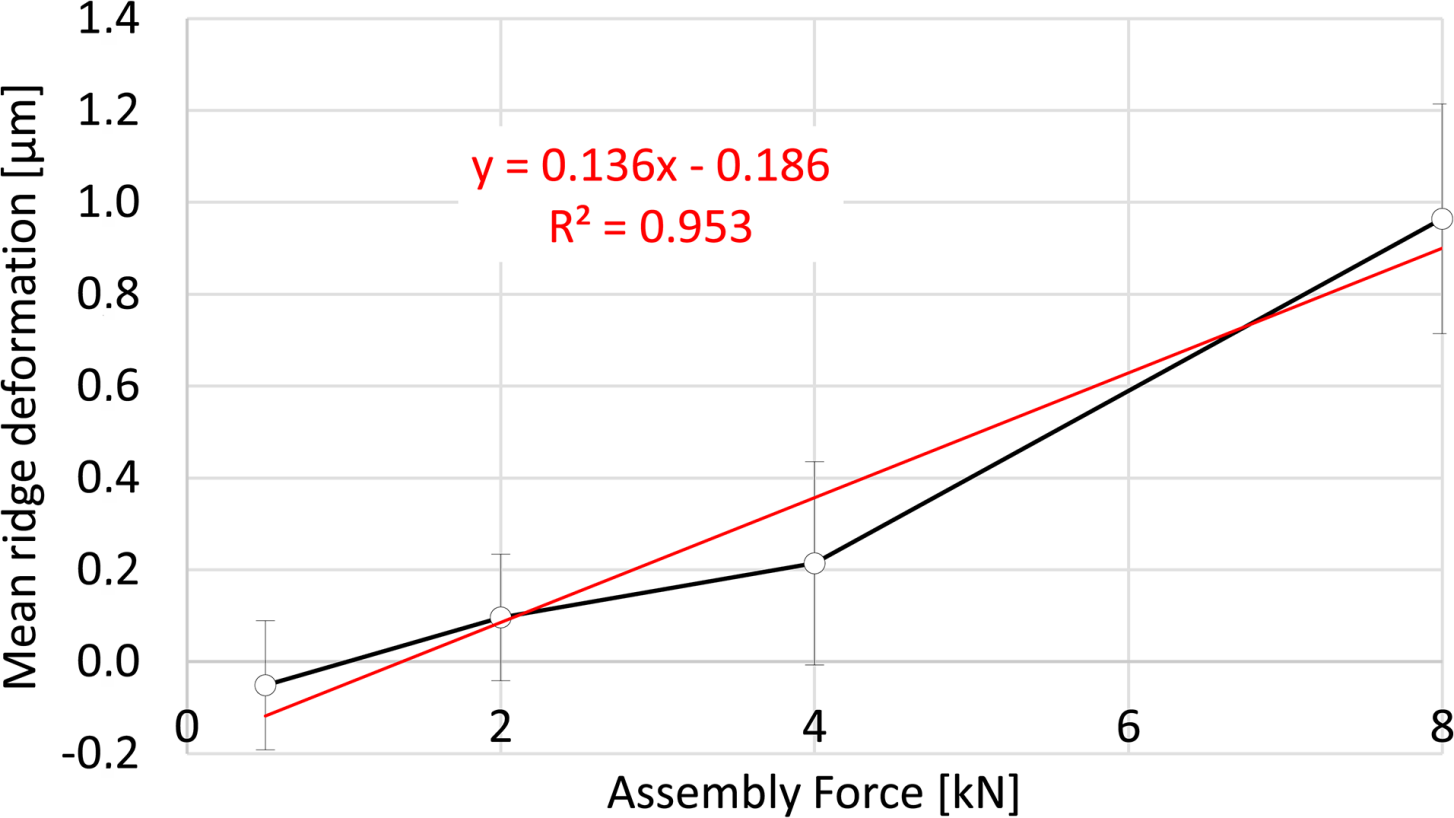
Mean ridge deformation. With increasing assembly force the mean ridge deformation increased.

The distribution of permanent deformation of the ridges was irregular along the length of the taper surface and varied between the three specimens according to the respective contact patterns (Fig 7). Not all of the ridges demonstrated permanent deformation, even for the highest assembly force of 8000 N (for which contact with every ridge was demonstrated). The maximum permanent deformation measured was 3.5 μm at 8000 N. Permanent deformation of the wave profile was characterised by flattening of the ridges, which resulted in a material expansion at the sides (Fig 8). Similarly to the increasing number of ridges in contact and their area of contact with increasing assembly forces, mean ridge deformation over the taper length also increased with increasing assembly force (Fig 9). For an assembly force of 500N, the total mean deformation (-0.05±0.14 μm) was below the measuring accuracy. An assembly force of 2000 N resulted in a mean permanent ridge deformation of 0.1±0.14 μm, an assembly force of 4000 N resulted in 0.21±0.22 μm and an assembly force of 8000 N resulted in 0.96±0.25 μm. The increase of mean permanent deformation of the ridges was linearly related to assembly force (R^2^ = 0.953, Fig 9). Significant differences in permanent ridge deformation were observed only for comparisons of 8000 N with lower assembly forces (p≤0.009).

## Discussion

This study addressed the post-operative contact condition of modular hip prostheses. Variable rates and extents of corrosion reported clinically and experimentally may be related to variable contact conditions. It was hypothesized that assembly forces modify the extent and distribution of the surface contact area at the taper interface between a cobalt chrome head and titanium taper with a threaded surface profile. This was supported using a new method to characterise local surface contact and permanent surface deformation.

The assembly forces applied in this study represent a clinical range [27]. Both the mean proportion of stem taper ridges in contact and the mean proportion of the contact surface, increased with increasing assembly force. Surface contact reached a maximum of 16.1% of the imaged surface area for the 8000 N assembly force, and affected as few as one of the 51 wave ridges for the lowest 500 N assembly force. Thus, contact is limited for this type of taper interface, even for very high assembly forces. Assembly forces of 4000 N or higher have been suggested to provide adequate function of the modular head-stem taper [28], while the low assembly force (500 N) represents a low intraoperative impaction force [27]. The low contact area is not necessarily mechanically deleterious, but the extent of surface contact required to achieve sufficient interface strength is unclear. Assuming a tight radial press fit of the surface profile ridges against the head taper surface, friction forces might be sufficient to prevent any relative motion of the region in contact, even under bending loads in patients. Furthermore, local contact at the open end (distal) might seal against fluid exchange with the joint capsule. Thus, for 8000 N assembly force all 51 wave ridges demonstrated contact, and for 4000 N, despite less than 100% contact of the ridges, contact was achieved at the proximal and distal ends in all three samples, which may ensure sealing. For the two lower assembly forces tested contact was not always achieved at the open end of the head, suggesting reduced resistance to fluid exchange.

The incidence of permanent deformation of the surface profile demonstrated that the elastic limit of the titanium alloy was exceeded locally and that maximal radial contact stresses were achieved in these regions. In this state the resistance to relative motion might be expected to be greatest, and thus desirable. Permanent radial deformation was not observed in measurable magnitudes for all ridges, even for the high (8000 N) assembly force (Fig 7). A very high assembly force would be expected to generate the safest situation, with the greatest resistance to relative motion. However, assembly forces of 8000 N or higher, cannot be applied with hammer blows due to the risk of bone fracture. It is noted that lower proportions of permanent deformation for lower assembly forces do not suggest a poor local press fit, but simply indicate that stresses remained within the elastic range of the material.

The particular modular combination tested in this study has been commonly implanted clinically [29,30]. Contact between the stem and head components will depend on the taper angle differences and the surface design. The three taper combinations had very similar taper angles between head and stem (Table 1), explaining the well-distributed contact observed along the length of the taper interface, even for the low assembly force. The varied distribution of contact and of permanent deformation reflect the variation in the height of the profile ridges along the length of the taper interface (Fig 2). It is assumed that the path measured is representative of the entire surface. Therefore, the irregular distribution of surface contact is to be expected clinically, becoming increasingly uniform as the local irregularities become compressed. Variable clinically applied assembly forces might provide an explanation for variations in clinical outcome for this design [31–32]. Other factors, such as contamination with particles or fluids [33–35], have been suggested to disrupt the taper interface. It is noted that, despite complete contact of the ridges for the highest assembly force, a continuous spiral channel through the thread grooves exists along the full contact length. To what extent this may allow fluid exchange remains to be investigated. The existence of fluid is unlikely to cause severe corrosion unless combined with mechanical mechanisms that remove the protective oxide layers of the metals [21]. It seems that the best chance of preventing disruption of the surface layer is to maximize the local radial press fit by applying high assembly forces and to ensure matching taper angles of the head and stem components.

The threaded stem surface profile was introduced to protect brittle ceramic heads from fracture, by distributing the assembly load and reducing local contact stresses. Ceramic component fracture rates of 0.004–0.35% have been reported [36–38]. Further development of alumina/zirconia composite ceramics has reduced the fracture probability further (e.g. BIOLOX delta®, 1:50,000) [39]. The surface profile of the stem has been maintained in some stem designs, which are now combined with metal heads. However, inclusion of a surface profile on the stem taper has been reported to increase the rate of corrosion of metal heads in comparison with smooth stem taper surfaces [25]. Since overload of metal heads is not an issue, their taper angle can be made more similar to that of the stem, in order to maximize contact area. Despite the identical stem and head taper angles used in the current study, contact was found to be irregular for low assembly forces, due to local surface irregularities (Fig 2). Retrievals at revision demonstrate preferential corrosion of the cobalt chrome head, with a clear mirrored imprint of the unchanged titanium stem taper surface profile [2,3,24]. Various patterns of corrosion have been observed in the cobalt chrome head [24], which may reflect the varied patterns of contact area observed for different implants under the varied assembly loads, demonstrated in the current study. Significant corrosion has been observed for titanium-on-titanium modular interfaces with matching taper angles and no surface profile [18]. Thus, whether or not the removal of the threaded surface profile from the stem taper design would decrease corrosion of the cobalt chrome head remains unclear and may depend on the manufacturing tolerances, which need be high enough to ensure uniform contact of a smooth surface.

The contact of smooth taper surface designs was not addressed in this study. It is quite possible that the actual proportion of contact at any interface is as low at that observed in the current study. This could be due to angular mismatches between the taper components at a macro level, or it could occur due to micro-roughness. As demonstrated in the current study and according to basic contact mechanics, the proportion of the interface actually in contact at any metal-metal interface would be assumed to increase with the applied assembly force. This is likely to be the case regardless of the surface geometry, topography or roughness. What will vary is the distribution of the actual contact area. These factors lead to specific corrosion patterns. The thread-like topography on the titanium stem taper tested in the current study leads to its own negative imprint in a mating cobalt chrome head clinically [24]. Experimentally, the corrosion rate of a cobalt chrome head on a titanium stem has been shown to decrease with increasing applied assembly force [20]. Both surface design and assembly force thus seem to play a role in corrosion patterns by influencing contact pressure and relative motion [40] and are therefore very likely to be related to contact patterns.

The method presented in the current study allows accurate measurement of surface contact and permanent deformation at a microscopic scale. Assessment of such implant surfaces for changes in geometry after clinical service was achieved using contact and non-contact measurements followed by a comparison with an assumed initial geometry [1,24,41]. However, surface contact between components tends to be assessed qualitatively, by visual observation of a coloured fat-based paste (for example, “Engineer’s Blue”) or ink, after assembly and disassembly of the interface. Such layers have a thickness that is heterogeneous, and low viscosity fluids were found in preliminary studies to run down into the troughs of the profile, causing a heterogeneous reflection and making automated digital imaging analysis difficult. The gold layer employed in the current analysis is uniform and thin (25.3 nm), relative to the profile to be measured. It can be easily and cheaply applied using standard electron microscopy equipment. The structure of the coating is columnar, radiating from the surface and ensuring some mechanical decoupling within the layer between loaded and unloaded regions. This prevents smearing and provides a clear delineation of regions where shear contact has occurred. Artefacts occurred because the surfaces could not be properly cleaned before scanning (to avoid disturbance of the gold layer) and due to light reflections during the scanning process. This is the reason for negative mean ridge deformations for lower assembly forces (500 N and 2000 N) which are within the precision of the measurement (Fig 9). It is noted that some gold transfer to the interface, and its influence on friction and seating in repeated tests with the same components cannot be ruled out. The surfaces were cleaned thoroughly with alcohol between experiments and no surface changes were observed by visual inspection.

The quantitative characterisation of the taper surface provided in this study is also an important input for mechanical models of the taper interface. Assuming that the corrosion observed clinically is related to local mechanical conditions, including shear contact and fluid transfer, local mechanical modelling will be essential in explaining taper corrosion patterns.

## Conclusions

In this study a new method was presented to characterise local interface contact conditions. This can be applied to any stem taper design and the effect of interface conditions, assembly forces and joint loading can be assessed. For components with similar head and stem taper angles, low assembly forces were observed to result in regions of limited contact. Even at relatively high assembly loads the actual contact area was found to be less than 20% of the overlapping interface area for a stem taper with a common threaded profile.
